# The Spliceosome Factor EFTUD2 Promotes IFN Anti-HBV Effect through mRNA Splicing

**DOI:** 10.1155/2023/2546278

**Published:** 2023-06-23

**Authors:** Pingping Hu, Yuwen Li, Wen Zhang, Rui Liu, Linya Peng, Ruirui Xu, Jinyuan Cai, Hui Yuan, Tiantong Feng, Anran Tian, Ming Yue, Jun Li, Wenting Li, Chuanlong Zhu

**Affiliations:** ^1^Department of Infectious Disease, The First Affiliated Hospital of Nanjing Medical University, Nanjing, China; ^2^Department of Pediatrics, The First Affiliated Hospital of Nanjing Medical University, Nanjing, China; ^3^Department of Infectious and Tropical Diseases, The Second Affiliated Hospital of Hainan Medical University, Hainan, China

## Abstract

**Methods:**

Using a CRISPR/Cas9 gene-editing system, *EFTUD2* single allele knockout HepG2.2.15 cells were constructed. Subsequently, the HBV biomarkers in *EFTUD2^+/–^* HepG2.2.15 cells and wild-type (WT) cells with or without IFN-*α* treatment were detected. And the EFTUD2-regulated genes were then identified using mRNA sequence. Selected gene mRNA variants and their proteins were examined by qRT-PCR and Western blotting. To confirm the effects of EFTUD2 on HBV replication and IFN-stimulated gene (ISG) expression, a rescue experiment in *EFTUD2^+/–^* HepG2.2.15 cells was performed by EFTUD2 overexpression.

**Results:**

IFN-induced anti-HBV activity was found to be restricted in *EFTUD2^+/–^* HepG2.2.15 cells. The mRNA sequence showed that EFTUD2 could regulate classical IFN and virus response genes. Mechanistically, *EFTUD2* single allele knockout decreased the expression of ISG-encoded proteins, comprising Mx1, OAS1, and PKR (EIF2AK2), through mediated gene splicing. However, EFTUD2 did not affect the expression of Jak-STAT pathway genes. Furthermore, EFTUD2 overexpression could restore the attenuation of IFN anti-HBV activity and the reduction of ISG resulting from *EFTUD2* single allele knockout.

**Conclusion:**

*EFTUD2*, the spliceosome factor, is not IFN-inducible but is an IFN effector gene. EFTUD2 mediates IFN anti-HBV effect through regulation of gene splicing for certain ISGs, including *Mx1*, *OAS1*, and *PKR*. EFTUD2 does not affect IFN receptors or canonical signal transduction components. Therefore, it can be concluded that EFTUD2 regulates ISGs using a novel, nonclassical mechanism.

## 1. Introduction

More than 350 million people around the world are chronically infected with the hepatitis B virus (HBV), which poses a serious threat to human health. Chronic carriers of HBV are at increased risk of developing cirrhosis and hepatocellular carcinoma [1]. Interferon *α* (IFN-*α*) is one of the leading antiviral agents for the treatment of chronic hepatitis B (CHB) [2]. The antiviral role of type I IFN is cascaded through binding to the type I IFN receptor (IFNAR1), stimulating the Janus tyrosine kinase (JAK) signal transducer and transcription activator pathway (STAT) [3], and then promoting the expression of genes stimulated by IFN (ISG). Among the “classical ISGs” myxovirus resistance gene 1 (*Mx1*), 2,5-oligoadencylate synthase (*OAS*) and RNA-dependent protein kinase (*PKR*) are the most significant ones, which have been found to mount antiviral activity against HBV and other viruses [4, 5]. Despite significant advances in treating hepatitis B, the response rate for IFN treatment in CHB patients is still unsatisfactory.

In our earlier studies, the elongation factor Tu GTP binding domain-containing protein 2 (EFTUD2), a GTPase spliceosome with a regulatory role in catalytic splicing and postsplicing complex disassembly, was shown to inhibit HCV infection by stimulation of the innate immune response [6]. Mechanistically, EFTUD2 exerted its antiviral activity by promoting ISG expression in HCV cell culture models [7]. Additionally, we discovered that a single nucleotide polymorphism (SNP), rs3809756, in the *EFTUD2* gene was associated with susceptibility to HBV infection [8]. In light of all of this, we proposed the hypothesis that EFTUD2 regulates IFN-induced ISG expression against HBV infection.

This study found a previously unrecognized function of EFTUD2 in regulating the IFN-induced anti-HBV effect. Besides, it was also confirmed that EFTUD2, the spliceosome factor, promotes the IFN-induced anti-HBV effect through ISG mRNA splicing. The present study reveals the positive role of EFTUD2 in IFN-*α* treatment of CHB, which could be used as a potential biomarker to predict the outcome of IFN treatment in HBV infection.

## 2. Materials and Methods

### 2.1. Cell Culture

We maintained HepG2.2.15 cells (GenBank: U95551), containing the full-length HBV genome and capable of producing replicative intermediates and mature virions in Dulbecco's Modified Eagle's Medium (DMEM, Thermos Fisher) supplemented with 10% fetal bovine serum (Gibco), 100 U/mL penicillin, and 0.1 mg/mL streptomycin at 37°C in a moist atmosphere with 5% CO_2_. For selection, G418 (0.4 mg/mL) was added to DMEM.

### 2.2. CRISPR/Cas9 for *EFTUD2* Gene Editing

A tool given by Zetsche et al. [9] was used to create short guide RNAs that targeted exon3 of the EFTUD2–202 transcripts (Table 1) based on the *EFTUD2* gene sequence in the NCBI database. As shown in Supplemental Figure 1A, the clustered regularly interspaced short palindromic repeats/CRISPR-associated protein 9 (CRISPR/Cas9) subtype II system, including the sgRNA oligo, Cas9, and protospacer adjacent motif (PAM), was associated with PX458-GFP vector [10]. Electroporation of the Px458-GFP-EFTUD2-sgRNA plasmid was performed on HepG2.2.15 cells. GFP-positive cells were selected after 48 h of transfection by fluorescence-activated cell sorting (FACS) (Supplemental Figure 1B). After the limiting dilution, the genetically modified cells were checked for monoclonal mutant cells. After that, we kept 43 monoclonal cells. DNA isolated from modified cells was amplified by PCR using specific identifying primers (Table 2). Electrophoresis of the PCR product on agarose gel revealed that the 39th strain was heterozygous (*EFTUD2*^+/-^). Other strains are wild type (WT), whereas the 38th and 40th strains were hybrid clones (Supplemental Figure 1C). The PCR products were sequenced, and the findings showed that one chromosome from the 39th strain had a WT sequencing length of 702 base pairs. There was a loss of 127 bp between the 410th and 537th bases of exon3 on another chromosome (Supplemental Figure 2).

### 2.3. ECIIA for the Detection of HBsAg and HBeAg

WT and *EFTUD2*^+/-^ HepG2.2.15 cells were seeded into 6-well plates at a density of 5 × 10^5^ cells per well and maintained in DMEM with or without IFN*α*-2b. HBsAg and HBeAg were detected by electrochemiluminescence immunoassay (ECIIA) kits (Roche, Switzerland). The optical density (OD) reading was evaluated with an automatic e602 electrochemical luminescence immune analyzer (Roche, Cobas, Switzerland).

### 2.4. Real-Time PCR for HBV DNA Quantification

WT and *EFTUD2*^+/-^HepG2.2.15 cells were seeded into 6-well plates at a density of 5 × 10^5^ cells per well and maintained in DMEM with or without IFN*α*-2b. We collected the supernatants after nine days of continuous treatment and detected the secretion of HBV DNA according to the quantitative detection kit of hepatitis B virus nucleic acids (Dan Gene Company, Guangzhou, China), following the manufacturer's protocol.

### 2.5. Immunofluorescence Assay for HBcAg Detection

We fixed the cells grown on cover glass coated with 0.1 mg/mL collagen and 4% paraformaldehyde (Wako) for 15 min and permeabilized them for 10 min with 0.2% Triton X-100. Cells were blocked with 10% goat blood serum for 60 min at 37°C and then treated with mouse-anti-HBc antibody (1 : 200 diluted, Abcam) overnight at 4°C. The treated cells were washed three times with PBS before incubating with a goat anti-mouse Alexa Fluor 594 dye-conjugated antibody (Jackson Immune Research, USA) at 37°C for 50 min. The resultant cells were then washed three times with PBS and counterstained with DAPI (Sigma) at room temperature. Fluorescent images were obtained using fluorescent microscopy (ZEISS, Germany) with 20x objective. ZEN light edition was used to analyze the images. The image shown represents three different microscopic fields; the percentage of HBcAg-positive cells was indicated (mean ± SD).

### 2.6. RNA Sequencing

After a nine-day treatment with IFN*α*-2b, total RNA (2 *μ*g) in WT and *EFTUD2*^+/-^HepG2.2.15 cells was extracted. Following the extraction of total RNA, the eukaryotic mRNA was enriched by Oligo (dT) beads. Subsequently, the enriched mRNA was fragmented into short fragments using fragmentation buffer and reverse-transcribed into cDNA by using NE Next Ultra RNA Library Prep Kit for Illumina (NEB #7530, New England Biolabs, Ipswich, MA, USA). The purified double-stranded cDNA fragments were end-repaired, base added, and ligated to Illumina sequencing adapters. AMPure XP beads (1.0x) were used to purify the ligation reaction. Ligated fragments were subjected to size selection by agarose gel electrophoresis and PCR amplified. The resultant cDNA library was sequenced using Illumina Novaseq6000 by Gene DiNovo Biotechnology Co. (Guangzhou, China). The Broad Institute (Cambridge, MA) uses a large-scale, automated variant of the Illumina TruSeq™ RNA Sample Preparation protocol for non-strand-specific RNA sequencing.

RNA differential expression analysis was carried out by DESeq2 software. Those genes/transcripts with a parameter of false discovery rate (FDR) below 0.05 (FDR < 0.05) and an absolute fold change greater than 2 (|log2FC| > 1) were considered differentially expressed genes. To identify whether a set of genes in specific GO terms/Reactome pathways/DO terms shows significant differences in two groups, we performed gene set enrichment analysis using software Gene Set Enrichment Analysis (GSEA) and MSigDB. Briefly, we input the gene expression matrix and classify genes using the signal-to-noise normalization method. Enrichment scores and *P* value were calculated using default parameters.

### 2.7. Western Blotting Analysis

The protein was collected, isolated, and transported to nitrocellulose membranes. And the concentration of each sample was measured using BCA Protein Assay Kit P0012S (Beyotime, China). Proteins were separated by using stacking gel and SDS-PAGE with a Tris-glycine system at 100 V for 90 min and transferred to polyvinylidene fluoride membranes (Millipore, USA). The membranes were then blocked by using 3% nonfat dry milk in phosphate-buffered with nonfat dry milk. After incubating the specific primary antibodies at 4°C for one night, the membranes were washed three times and incubated with horseradish peroxidase- (HRP-) conjugated secondary antibody. Band intensities were quantified by the image Image Lab software.

The antibodies (Abs) including EFTUD2 (1 : 5,000 diluted), PKR (1 : 5,000 diluted), and Mx1 (1 : 2000 diluted) were bought from Abcam. Abs against OAS1 (diluted at 1 : 200) were acquired from Santa Cruz. And Abs against STAT1 (diluted at 1 : 1000) and p-STAT1 (diluted at 1 : 1000) were purchased from Cell Signaling. The primary antibody against *β*-actin and the secondary antibody were obtained from Bioss Company in Beijing, China (diluted at 1 : 1000 and 1 : 10000, respectively).

### 2.8. RT-qPCR for ISG Detection

RNA was extracted using TRIzol (Invitrogen, Carlsbad, CA) reagent and reverse-transcribed into cDNA with PrimeScript RT Master Mix (Takara, Dalian, China). RNA expression levels were measured by qRT-PCR performed on a 7500 HT qRT-PCR system (Applied Biosystems, Life Technologies, Darmstadt, Germany) using the CT method. The relative mRNA levels of all target genes were normalized to the level of the house-keeping gene glyceraldehyde 3-phosphate dehydrogenase (GAPDH).  Table 3 shows the primer sequences for target genes.

### 2.9. siRNA and Plasmid Transfection

siRNAs (small interfering RNA) and plasmids were transfected into cells, respectively, by Lipo3000 (Beyotime, China) following the manufacturer's instructions. The *EFTUD2* gene was knocked down using si-EFTUD2, and a nontargeting negative siRNA (si-Neg) served as a control (Table 4). P-EFTUD2 (pCMV3-GFP-Puro-EFTUD2-tv2, pEFTUD2) was used for the overexpression of EFTUD2, and p-NC (pCMV3-GFP-Puro-NC, nontargeting control plasmids) was used as a control. All of these siRNAs and plasmids were manufactured by the GenePharma Biotech Company (Shanghai, China). Western blotting was used to confirm gene expression levels.

### 2.10. Statistical Analysis

Statistical analysis was performed using a two-tailed Student's *t*-test. Unless otherwise stated, data are represented as mean ± SD (standard deviation) of at least three sample replicates.

## 3. Results

### 3.1. *EFTUD2* Single Allele Knockout Cell Model

To investigate the function of EFTUD2 on the IFN anti-HBV effect, we attempted to knockout the *ETFUD2* gene in HepG2.2.15 cells by CRISPR/Cas9 gene-editing technology. The HepG2.2.15 cells were transfected with the Px458-GFP-EFTUD2-sgRNA plasmid and harbored monoclonal mutant cells. We next studied the mutational status of the two alleles in individual cell clones and found that most of the clones were WT. There was only one *EFTUD2* heterozygous clone, and homozygous was difficult to survive. This may be due to the fact that *EFTUD2* was significant for cell survive. Completely deleted of *EFTUD2* gene may lead to cell death. This finding is consistent with a study on neuronal apoptosis in a TALEN-induced *EFTUD2* mutant in zebrafish [11].

Sequencing alignment revealed that *EFTUD2* heterozygous HepG2.2.15 cells (*EFTUD2^+/–^* HepG2.2.15 cells) with monoallelic sequence had a 127 bp deletion between the 410th base and the 537th base in exon3 (Supplemental Figure 2).

**Figure 1:**
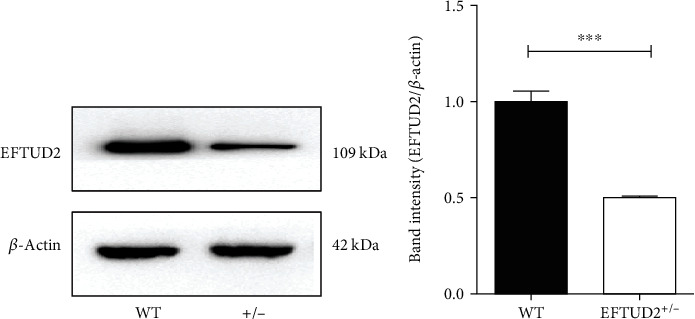
EFTUD2 single allele knockout cell model. (a) The expression of EFTUD2 protein in EFTUD2^+/-^HepG2.2.15 cells and WT HepG2.2.15 cells was detected by Western blotting. (b) Band intensities were quantified by the image Image Lab software. Mean ± SD from three independent experiments was shown. Student's *t*-test was used to determine the significance of differences between the two groups. ^∗^*P* < 0.05, and ^∗∗^*P* < 0.01.

To observe the consequence of the engineered deletion of exon3, we compared the levels of EFTUD2 protein in WT and *EFTUD2* heterozygous (*EFTUD2*^+/–^) HepG2.2.15 cells. Western blotting analysis with an EFTUD2 antibody indicated a statistically significant 50.17% reduction in EFTUD2 protein levels in *EFTUD2*^+/–^ cells than WT (*t*-test, *P* < 0.05) (Figure 1). Therefore, we concluded that levels of EFTUD2 protein were reduced in *EFTUD2*^+/–^ HepG2.2.15 cells with deletion of exon3. And then, we used this cell model in the following experiment.

### 3.2. IFN Anti-HBV Activity Was Compromised in *EFTUD2^+/-^*HepG2.2.15 Cells

**Figure 2:**
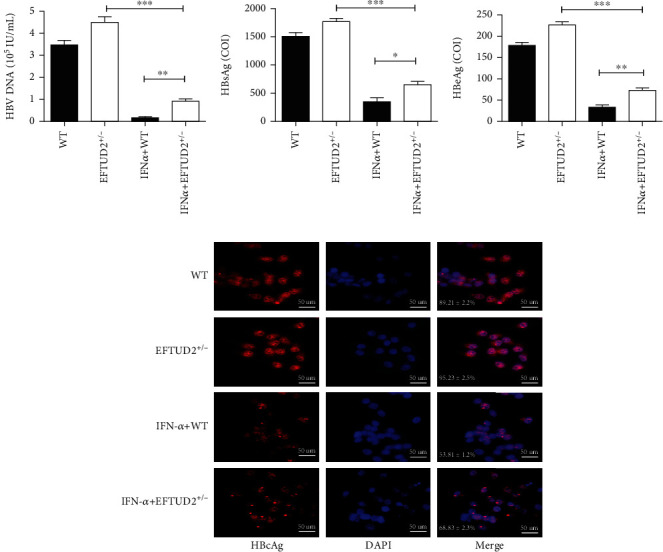
IFN anti-HBV activity was restricted in EFTUD2^+/-^HepG2.2.15 cells. WT and EFTUD2^+/-^HepG2.2.15 cells were maintained in DMEM with or without IFN*α*-2b (5000 IU/mL) for 9 days. (a–c) The supernatants were then collected. HBV DNA was evaluated by real-time PCR, and HBeAg and HBsAg were detected by ECIIA. Mean ± SD from three independent experiments was shown. Student's *t*-test was used to determine the significance of differences between the two groups. ^∗^*P* < 0.05, and ^∗∗^*P* < 0.01. (d) Cells were immunostained with antibodies against HBV capsid protein (HBcAg) and visualized under fluorescence microscopy (20x). Nuclei were counterstained with DAPI. The image shown represents three different microscopic fields; the percentage of HBcAg-positive cells was indicated (mean ± SD).

To examine the role of EFTUD2 in IFN-mediated anti-HBV activity, HBV DNA replication, HBeAg, HBsAg secretion, and HBcAg expression were identified in WT and *EFTUD2^+/-^* HepG2.2.15 cells before and after IFN treatment. As shown in Figure 2, HBV DNA as well as HBeAg and HBsAg and the percentage of HBcAg-positive cells were significantly decreased after nine days [12] of continuous treatment using 5000 IU/mL IFN*α*-2b (IFN-*α* concentration was selected in Supplemental Figure 4), in both WT and *EFTUD2^+/-^* HepG2.2.15 cells, while *EFTUD2* single allele knockout displayed 5.43-fold increase in HBV DNA (*P* < 0.01), 2.80-fold increase in HBsAg (*P* < 0.05), and a 3.29-fold increase in HBeAg (*P* < 0.05) as compared to WT HepG2.2.15 cells (Figures 2(a)–2(c)). Meanwhile, the percentage of HBcAg-positive cells was 15% higher (*P* < 0.01) in *EFTUD2*^+/-^ HepG2.2.15 cells compared to WT HepG2.2.15 cells after IFN treatment (Figure 2(d)).

Our findings suggested that IFN*α*-2b significantly inhibited HBV DNA replication, as well as HBeAg and HBsAg secretion and HBcAg expression. The single allele knockout of *EFTUD2* not only increased HBV replication but also compromised IFN antiviral activity, demonstrating that EFTUD2 exerts IFN-associated antiviral activity in the HBV replication cell model.

### 3.3. Differential Gene Expression Analysis Using mRNA-seq

To further investigate the mechanism of EFTUD2 regulating IFN-associated antiviral activity, a transcriptome RNA sequencing was performed in WT and *EFTUD2^+/-^*HepG2.2.15 cells after IFN*α*-2b treatment. From the mRNA-seq data, we obtained sequence reads for 20323 genes (Supplemental Table 1). A total of 814 genes showed greater than 2-fold differences (FDR < 0.05 and |log2FC| > 1), including 439 genes upregulated and 371 genes downregulated by *EFTUD2* single allele knockout (Supplemental Table 2). According to the GO and Reactome of gene enrichment analyses, EFTUD2 controls multiple functional pathways, comprising immune response and negative regulation of viral genome replication (Supplemental Figure 3). Genes in that enriched pathway comprised classical IFN and virus response genes such as *Mx1*, *OAS1*, and *PKR* (*EIF2AK2*). DO analysis also indicated that the genes were related to hepatitis B.

### 3.4. EFTUD2 Promoted the Expression of IFN-*α* Downstream Antiviral Effectors

**Figure 3:**
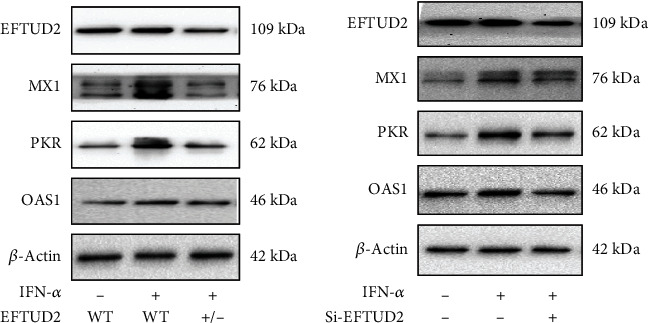
Decreased expression of EFTUD2 suppresses the expression of IFN-*α* downstream antiviral effectors. (a) Both EFTUD2^+/-^HepG2.2.15 cells and WT HepG2.2.15 cells were treated with 5000 IU/mL IFN-*α* for 24 h. IFN-*α*-induced Mx1, OAS1, and PKR expression levels were measured by Western blotting. (b) HepG2.2.15 cells were transfected with si-EFTUD2 or si-NC for 24 h and then treated with 5000 IU/mL IFN-*α* for 24 h. The mRNA protein levels of Mx1, OAS, and PKR were analyzed by Western blotting after 48 h of transfection. The experiments were repeated three times each.

*Mx1*, *OAS*, and *PKR* are classical downstream ISGs, having antiviral properties. In order to exert antiviral activity, these ISG-encoded proteins interfere with certain phases of viral replication or trigger the degradation of viral RNAs and proteins [5, 13–15]. The expression of MxA, OAS, and PKR induced by IFN in WT and *EFTUD2^+/-^* HepG2.2.15 cells was examined by Western blotting to determine the role of EFTUD2 in modulating these downstream IFN effectors. IFN-induced Mx1, OAS1, and PKR expressions in *EFTUD2^+/-^*HepG2.2.15 cells were determined to be significantly lower than in WT HepG2.2.15 cells (Figure 3(a)). Furthermore, we observed that IFN-*α* treatment had no effect on *EFTUD2* mRNA expression. Overall, these findings showed that single allele knockout of *EFTUD2* reduces IFN antiviral efficacy by suppressing ISG expression.

In the meantime, HepG2.2.15 cells were transfected with si-EFTUD2 (EFTUD2 small interference RNA) and si-NC (control siRNA not targeting), and the transfected cells were treated with 5000 IU/mL IFN-*α* for 24 h. Subsequently, the expression of the Mx1, OAS1, and PKR proteins was measured. The findings indicated that EFTUD2 interference decreased the expression of Mx1, OAS1, and PKR (Figure 3(b)). Therefore, it was confirmed that silencing EFTUD2 abrogates the antiviral activity of IFN by negatively regulating the expression of ISGs.

### 3.5. EFTUD2 Upregulated Mx1, OAS1, and PKR Expressions by Pre-mRNA Splicing

EFTUD2 is a core component of the spliceosome and has recently been identified as a novel regulator of innate immunity [16]. Based on our previous study, EFTUD2 regulates the synthesis of RIG-I and MDA5 through mRNA splicing [7]. So we next sought to investigate whether its absence results in alterations in pre-mRNA splicing. We then used the rMATS computational package to identify splicing differences between WT and EFTUD2^+/-^HepG2.2.15 cells after IFN-*α* treatment. And we found that EFTUD2 single allele knockout affects IFN-induced *MX1*, *OAS1*, and *PKR* transcript frequencies. As a result, the protein coding transcripts like *MX1-201* (ENST00000288383), *OAS1-202* (ENST00000445409), and *EIF2AK2-203* (ENST00000405334) decreased, and nonprotein coding transcripts like *MX1-210* (ENST00000467510), *OAS1-205* (ENST00000549820), and *EIF2AK2-205* (ENST00000462861) increased in EFTUD2^+/-^HepG2.2.15 cells (Supplemental table 3).

**Figure 4:**
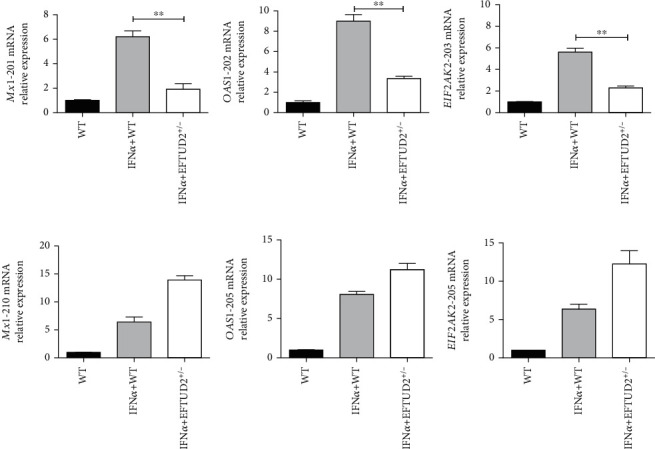
EFTUD2 upregulated *Mx1*, *OAS1*, and *PKR* expressions by pre-mRNA splicing. Both EFTUD2^+/-^HepG2.2.15 and WT HepG2.2.15 cells were treated with 5000 IU/mL IFN-*α* for 8 h. We compared *Mx1*, *OAS1*, and *PKR* transcript mRNA expressions between EFTUD2^+/-^HepG2.2.15 cells and WT HepG2.2.15. (a) *Mx1*-201 mRNA levels. (b) *OAS1*-202 mRNA levels. (c) *EIF2AK2*-203 mRNA levels. (d) *Mx1*-210 mRNA levels. (e) *OAS1*-205 mature mRNA levels. (f) *EIF2AK2-205* mRNA levels. *Mx1*-201, *OAS1*-202, and *EIF2AK2*-203 are protein encoding transcripts, and *Mx1*-210, *OAS1*-205, and *EIF2AK2-205* are nonprotein encoding transcripts based on Ensembl bioinformatics. Mean ± SD from three independent experiments performed in triplicate is shown. Student's *t*-test was used to determine the statistical difference between the two groups. ^∗^*P* < 0.05, and ^∗∗^*P* < 0.01.

To verify differential splicing by rMATS analysis, we performed qPCR to compare the selected transcripts in IFN (5000 IU/mL, 8 h) induced WT and *EFTUD2^+/-^* HepG2.2.15 cells. *MX1-201* mRNA levels in WT cells were found to be 3.23 times higher than *EFTUD2^+/-^*HepG2.2.15 cells (*P* < 0.01) (Figure 4(a)). The levels of *OAS1-202* mRNA in WT HepG2.2.15 cells were 2.68 times higher than those of *EFTUD2^+/-^*HepG2.2.15 cells (*P* < 0.01) (Figure 4(b)). The levels of *EIF2AK2-203* (*PKR*) mRNA in WT HepG2.2.15 cells were 2.44-fold greater than *EFTUD2* single allele knockout cells (P < 0.01) (Figure 4(c)). At the same time, the levels of the *MX1-210*, *OAS1-205*, and *EIF2AK2-205* in WT HepG2.2.15 cells were lower than *EFTUD2^+/-^*HepG2.2.15 cells (Figures 4(d)–4(f)).

*EFTUD2* single allele knockout influenced the frequency of IFN-induced *Mx1*, *OAS1*, and *PKR* variant mRNA expressions, confirming EFTUD2 upregulated *Mx1*, *OAS1*, and *PKR* expressions by pre-mRNA splicing. These results demonstrated that EFTUD2 mediates IFN anti-HBV effect through regulation mRNA splicing for certain ISGs.

### 3.6. EFTUD2 Regulation of Classical ISGs Did Not Act through Direct Regulation of the Jak-STAT Pathway

**Figure 5:**
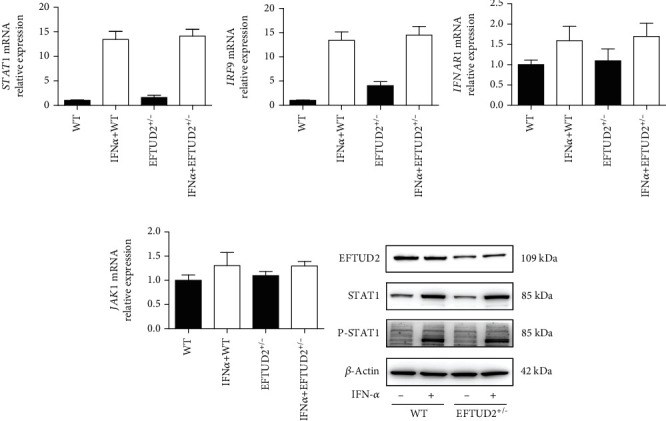
EFTUD2 regulation of classical ISGs did not act through direct regulation of the JAK-STAT pathway. (a–d) RT-qPCR detected the STAT1, IRF9, IFNAR1, and JAK1 mRNA in WT and EFTUD2^+/-^HepG2.2.15 cells before and after IFN treatment. (e) After incubated with 5000 IU/mL IFN-*α* for 24 h, STAT1 and STAT1 phosphorylation (P-STAT1) were assessed by Western blotting in WT and EFTUD2^+/-^HepG2.2.15 cells.

The antiviral role of type I IFN is cascaded *via* binding to type I IFN receptor (IFNAR1), stimulating the JAK/STAT1 pathway, and leading to dimerization of STAT1 and STAT2 in the association of IFN regulatory factor 9 (IRF9) to form IFN-stimulated gene factor 3 complex, which translocates to the nucleus and binds to ISRE to induce transcription of ISGs [17]. Consequently, we evaluated the relationship between EFTUD2 and the canonical IFN-induced JAK/STAT1 pathway and found that single allele knocking out *EFTUD2* did not affect *STAT1*, *IRF9*, *IFNAR1*, and *JAK1* mRNA expressions (Figures 5(a)–5(d)). Western blot analysis further indicated that *EFTUD2* single allele knockouts did not decrease phosphorylation of STAT1 in HepG2.2.15 cells with or without IFN-*α* treatment (Figure 5(e)). The above findings indicate that EFTUD2 does not affect IFN receptors or canonical signal transduction components in JAK-STAT pathway.

### 3.7. ISGs Reduced Can Be Rescued by EFTUD2 Overexpression

**Figure 6:**
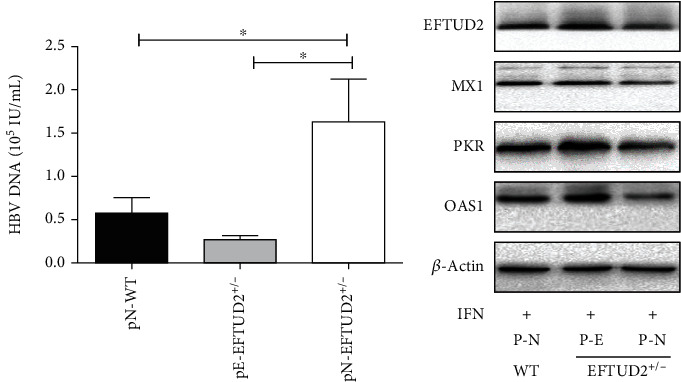
EFTUD2 single allele knockout results in ISGs reduced can be rescued by EFTUD2 overexpression. EFTUD2^+/-^HepG2.2.15 cells were transfected with EFTUD2 plasmid and negative control plasmid for 24 h. WT EFTUD2 cells were used as control and then treated with 5000 IU/mL IFN-*α* for 24 h. (a) HBV DNA was detected by qPCR 48 h after transfection. (b) 48 h after transfection, the protein levels of MxA, OAS1, and PKR were analyzed by Western blotting. Mean ± SD from three independent experiments performed in triplicate is shown. Student's *t*-test was used to determine the statistical difference between the two groups.

To further confirm *EFTUD2* single allele knockout compromised IFN anti-HBV activity through downregulating ISG expression, a rescue experiment in *EFTUD2^+/-^*HepG2.2.15 cells was carried out with p-EFTUD2 plasmids (pE). In this study, HBV DNA and ISG-encoded proteins in pE-*EFTUD2^+/-^*cells, pN-*EFTUD2^+/-^* (nontargeting control plasmids) cells, and pN-WT HepG2.2.15 cells were identified after treatment with IFN-*α*.  Figure 6(a) shows that the overexpression of EFTUD2 decreased the level of HBV DNA in pE-EFTUD2^+/-^HepG2.2.15 cells than pN-*EFTUD2*^+/-^HepG2.2.15 cells (*P* < 0.05). And the ISG-encoded proteins in pE-*EFTUD2^+/-^*HepG2.15 cells were significantly higher than in pN-*EFTUD2^+/-^*HepG2.2.15 cells (Figure 6(b)). Based on these findings, we determined that overexpression EFTUD2 in *EFTUD2^+/-^*HepG2.2.15 cells restored the impaired antiviral activity of IFN as well as the reduction of ISGs due to *EFTUD2* single allele knockout.

## 4. Discussion

IFN is one of the primary antiviral agents for the treatment of CHB. Unfortunately, the treatment response rate is far from satisfactory [18]. Host factors have long been considered to affect the efficacy of IFN therapy in patients with CHB [19, 20]. Finding the relation of the host gene to IFN response is immediately needed for CHB treatment. EFTUD2 is a constituent of the U5 snRNP in the spliceosome that facilitates pre-mRNA splicing [21, 22]. EFTUD2 has been implicated in various development processes, such as myofilament organization and the development of P granule development [23, 24]. Mutations in EFTUD2 lead to craniofacial abnormalities, such as mandibulofacial dysostosis and esophageal atresia [25, 26]. By using a functional genomic screen, a previously study shown that mRNA processing is essential for the impact of IFN-induced anti-HCV [27]. Furthermore, *EFTUD2* was identified as a new host gene that mediates IFN-*α*'s effects against HCV [7]. Moreover, our earlier work found that an SNP, rs3809756, in the *EFTUD2* gene was significantly associated with susceptibility to HBV infection [8]. To further explore the possible roles of EFTUD2 in IFN-*α*'s anti-HBV effect, the present study used HepG2.2.15 cells, the HBV replication cell culture model, to explain the mechanism of response to IFN and identify potential biomarkers to help predict treatment results.

Using the *EFTUD2^+/-^*HepG2.2.15 cell model, we first determined that *EFTUD2* single allele knockout would compromise IFN anti-HBV activity. These findings suggest that EFTUD2 is an IFN-associated antiviral host gene against HBV. This scenario supports the hypothesis that EFTUD2 plays an important role in IFN-*α* response and can act as a biomarker. We also used mRNA-seq to identify RNAs that EFTUD2 regulates to better understand the mechanism of EFTUD2 involved in IFN-associated antiviral activity. According to the GO and Reactome of gene enrichment analyses, EFTUD2 controls several functional pathways, including immune response and negative regulation of viral genome replication. The differentially expressed genes in that enriched pathway included classical ISGs like *Mx1*, *OAS1*, and *PKR*. Therefore, we proposed that EFTUD2 mediates ISG expression to promote IFN-associated anti-HBV activity. The RNA-Seq bioinformatic differential analysis was then confirmed by qPCR and Western blotting of selected genes. *EFTUD2* single allele knockout was found to significantly decrease the expression of key ISGs, including *MxA*, *OAS*, and *PKR*, but not *STAT1*, *IRF9*, *IFNAR1*, or *JAK1*. Moreover, the reduced Mx1, OAS1, and PKR proteins can be rescued after EFTUD2 overexpression. Lastly, EFTUD2 single allele knockout influenced the frequency of IFN-induced Mx1, OAS1, and PKR variant mRNA expressions, as the protein coding transcripts (based on Ensembl bioinformatics.) decreased and nonprotein coding transcripts increased in EFTUD2^+/-^HepG2.2.15 cells, confirming that EFTUD2 upregulated ISG expression by pre-mRNA splicing. The present findings indicate that EFTUD2 regulation of classical ISGs is differentially expressed and independent of JAK-STAT activation.

**Figure 7:**
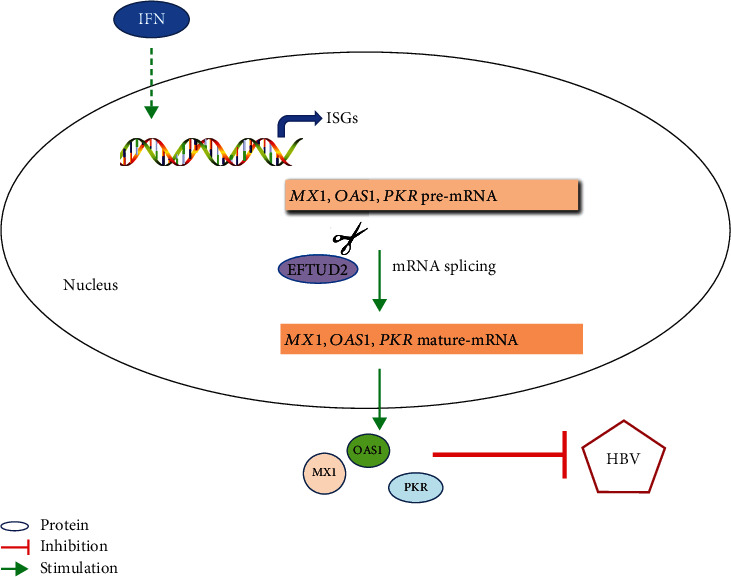
A proposed model for EFTUD2 in IFN-induced anti-HBV effect. The binding of IFN-*α* to type I IFN receptor activates the JAK-STAT pathway, ISRE transcription is initiated through signal transduction, and ISG expression is induced. EFTUD2 exerts its anti-HBV action through differential splicing of some ISGs, including Mx1, OAS1, and PKR. Furthermore, these antiviral proteins provide direct antiviral effects at various stages of the HBV life cycle.

In light of our findings, we propose a model for EFTUD2 regulating anti-HBV effect of IFN. The binding of IFN-*α* to type I IFN receptor activates the JAK-STAT pathway, and ISRE transcription starts through signal transduction; then, ISG expression is induced. Differential splicing of some ISGs, including *Mx1*, *OAS1*, and *PKR*, is responsible for EFTUD2's anti-HBV action. Moreover, these antiviral proteins provide direct antiviral effects at various stages of the HBV life cycle (Figure 7).

A number of intriguing things were also observed during the experiment. Homozygous *EFTUD2* knockout cells were difficult to obtain. Besides, the proliferation rate of *EFTUD2^+/-^*cells was lower than WT (Supplemental Figure 5). Hence, we speculated that the deletion of the *EFTUD2* gene would cause cell death. Mutations in the *EFTUD2* gene have been reported to cause p53-dependent apoptosis in neural progenitors of zebrafish [11]. And homozygous mutation of Eftud2 in neural crest cells causes craniofacial malformations and embryonic lethality [28]. In a similar study, the loss of function mutation of *EFTUD2* was reported to result in preimplantation arrest in the mouse. EFTUD2 is essential for postimplantation survival. According to these findings, EFTUD2 protein plays a significant role in cell life activities [29]. And there is no population-wide deletion of *EFTUD2* (*EFTUD2^−/−^*). However, in some cases, SNP mutations may result in lower EFTUD2 expression, which are difficult to eradicate HBV or insensitive to IFN therapy. Our preliminary research revealed that the EFTUD2 rs3809756 A to C mutation results in downregulation of EFTUD2 expression, further increasing host vulnerability to HBV infection and decreasing sensitivity to IFN therapy [8]. The cell model employed in this study can precisely imitate this situation and analyze the biological mechanisms of inadequate IFN therapy in EFTUD2 low expression patients.

In conclusion, the present study found that the variation of IFN-*α*'s anti-HBV response is related to differential *EFTUD2* gene expression levels, providing new insights into the EFTUD2-mediated regulation of ISGs. Mechanistically, EFTUD2 induces IFN anti-HBV effect through ISG mRNA splicing. Our study reveals that EFTUD2 contributes to IFN-*α* treatment of CHB and offers a potential biomarker to predict IFN treatment outcomes in HBV infection.

## Figures and Tables

**Table 1:** SgRNAs targeted to exon3 of EFTUD2 gene.

sgRNA	Sequence (5′⟶3′)
EFTUD2 sgRNA1-F EFTUD2 sgRNA1-R	CACCG GCACCACCTCCATCCCAGGG AAAC CCCTGGGATGGAGGTGGTGCC
EFTUD2 sgRNA2-F EFTUD2 sgRNA2-R	CACCG GCAGGTACATCAGGAGGACAT AAAC ATGTCCTCCTGATGTACCTGCC

**Table 2:** Primers used for validation of EFTUD2 sequence change.

Primers	Sequence (5′⟶3′)
EFTUD2-F EFTUD2-R	GTAAAGGGCTGTGCTGTGAT AACGACAGTCTTCATGCACC

**Table 3:** Primers used for RT-qPCR.

Gene target	Sequence (5′⟶3′)
GAPDH-F GAPDH-R	ACAGTCCATGCCATCACTGCC GCCTGCTTCACCACCTTCTTG
EFTUD2-F EFTUD2-R	CAATATCATGGACACTCCAGGAC CGGTCAATCTTGTTGATGCACA
Mx1-210-F (ENST00000467510) Mx1-210-R (ENST00000467510)	ACTACGACCGCAGAGCTGAACCTT AGGCCCCACTCCAGACCCACA
OAS1-205-F (ENST00000549820) OAS1-205-R (ENST00000549820)	ACCTACCCAAGGGCACATCACT GGTATGTCCCCAGTGCCCTATTCCC
EIF2AK2-205-F (ENST00000462861) EIF2AK2-205-R (ENST00000462861)	ACTCCAGCCTGGGGATCCTATCTCA GGGCAACTTCCAGTCCTGCAAGCTC
Mx1-201-F (ENST00000288383) Mx1-201-R (ENST00000288383)	CCCTTCCCAGAGGCAGCGGG CTGATTGCCCACAGCCACTC
OAS1-202-F (ENST00000445409) OAS1-202-R (ENST00000445409)	GGTGGTAAAGGGTGGCTCCTC TCTGCAGGTAGGTGCACTCC
EIF2AK2-203-F (ENST00000405334) EIF2AK2-203-R (ENST00000405334)	CCAGTGATGATTCTCTTGAGAGC CCCCAAAGCGTAGAGGTCCA

**Table 4:** Sequence of siRNAs.

siRNA	Sequence (5′⟶3′)
Si-negative control	UUCUCCGAACGUGUCACGUTT ACGUGACACGUUCGGAGAATT
Si-EFTUD2	CACCUUUGGUGACAUUAAUTT AUUAAUGUCACCAAAGGUGTT

## Data Availability

The original datasets generated or analyzed during the present study are available from the corresponding author.

## Supplementary Materials

Supplementary Materials Supplemental Figure 1: Establish ETFUD2^+/-^HepG2.2.15 cells. Supplemental Figure 2: Exon 3 of the EFTUD2 gene has 127 bp deletion between sgRNA1 and sgRNA2 cleavage sites. Supplemental Figure 3: EFTUD2-regulated functional pathways. Supplemental Figure 4: Choose the concentration of IFN*α*-2b. Supplemental Figure 5: Effects of EFTUD2 on cell proliferation. Supplemental Table 1: List of total gene identified by RNA-Seq differential analysis. Supplemental Table 2: list of selected gene for Gene Ontology (GO) analysis, Disease Ontology (DO) analysis, and Reactome analysis. Supplemental Table 3: The difference of spliced transcripts between WT and EFTUD2^+/-^ cells after IFN treatment.
