# Supplementary material for: The Spliceosome Factor EFTUD2 Promotes IFN Anti-HBV Effect through mRNA Splicing
Source: Mediators Inflamm. 2023 Jun 23;2023:2546278. doi: 10.1155/2023/2546278 (PMC10313468; doi:10.1155/2023/2546278)
Supplement: Supplementary Materials — Supplemental Figure 1: Establish ETFUD2+/-HepG2.2.15 cells. Supplemental Figure 2: Exon 3 of the EFTUD2 gene has 127 bp deletion between sgRNA1 and sgRNA2 cleavage sites. Supplemental Figure 3: EFTUD2-regulated functional pathways. Supplemental Figure 4: Choose the concentration of IFNα-2b. Supplemental Figure 5: Effects of EFTUD2 on cell proliferation. Supplemental Table 1: List of total gene identified by RNA-Seq differential analysis. Supplemental Table 2: list of selected gene for Gene Ontology (GO) analysis, Disease Ontology (DO) analysis, and Reactome analysis. Supplemental Table 3: The difference of spliced transcripts between WT and EFTUD2+/- cells after IFN treatment. [file 2546278.f1.zip › Supporting Online Material-0618.docx]

**Supporting Online Material for**

**The spliceosome factor EFTUD2 promotes IFN anti-HBV effect through mRNA splicing**

**Pingping Hu^1,^** **^a^, Yuwen Li^2,^** **^a^, Wen Zhang^1^, Rui Liu^3^, Linya Peng^1^, Ruirui Xu^1^, Jinyuan Cai^1^, Hui Yuan^1^, Tiantong Feng^1^, Anran Tian^1^, Ming Yue^1^, Jun Li^1^, Wenting Li^3^, Chuanlong Zhu^1, 3, *^**

*** Correspondence:** Department of Infectious Disease, the First Affiliated Hospital of Nanjing Medical University, 300 Guangzhou Rd., Nanjing 210029, China.

Tel: +86 25 6830 6375

Email: zhuchuanlong@jsph.org.cn

**Table of Contents for Supporting Online Material**

This file includes legends for Supplemental Materials and Methods, Supplemental Figure 1-5 and Supplemental Table 1-3.

Supplemental Figure 1. Establish *EFTUD2^+/-^*HepG2.2.15 cells.

Supplemental Figure 2. Exon 3 of the EFTUD2 gene has 127bp deletion between sgRNA1 and sgRNA2 cleavage sites.

Supplemental Figure 3. EFTUD2 regulated functional pathways.

Supplemental Figure 4. Choose the concentration of IFNα-2b.

Supplemental Figure 5. Effects of EFTUD2 on cell proliferation.

Supplemental Table 1. List of total gene identified by RNA-Seq differential analysis.

Supplemental Table 2. List of selected gene for Gene Ontology (GO) analysis, Disease Ontology (DO) analysis and Reactome analysis.

Supplemental Table3. The difference of spliced transcripts between WT and EFTUD2+/- cells after IFN treatment.

1. **Supplemental Materials and Methods**

**Cell counting kit-8 (CCK-8) assay**

HepG2.2.15 cells and EFTUD2+/-HepG2.2.15 cells were seeded in 96-well plates at a density of 1 × 10^4^ cells per well. After 24 h of IFN-α treatment, CCK-8 (Domino Laboratories, Rockville, MA) reagent was added (10 ul/well) and incubated for 3 h. Following vortexing for 10 min, the absorbance value (OD) of each well was measured at 450 nm. Each sample was tested in five repeats in three separate experiments. Survival rate (%) = [(OD experimental-OD blank)/ (OD negative control-OD blank) x 100%.

**Differential splicing Analysis**

The software rMATS (version 4.0.1) (http://rnaseq-mats.sourceforge.net/index.html) was used to identify differential splicing events between WT and EFTUD2+/-HepG2.2.15 cells after IFNα-2b treatment. We identified AS events with a false discovery rate (FDR) <0.05 in a comparison as significant AS events.

1. **Supplemental Figures**

**Supplemental Figure 1. Establish *EFTUD2^+/-^*HepG2.2.15 cells.** (A) Two CRISPR guide RNAs (CRISPR guide RNA 1/CRISPR guide RNA 2, red letters) were designed to bind in exon 3 in the EFTUD2-202 transcript. Target sequences are highlighted in green letters. PAM sequences are highlighted in purple letters. (B) GFP-positive cells were selected by FACS. (C) The results of agarose gel electrophoresis indicated that the 39th strain was heterozygous (*EFTUD2^+/-^*). The 38th and 40th strains were hybrid clones. Others are WT.

**Supplemental Figure 2.** Exon 3 of the EFTUD2 gene has 127bp deletion between sgRNA1 and sgRNA2 cleavage sites. (A) PCR products sequence of two chromosomes. (B) PCR products sequence of two chromosomes. The results showed that one chromosome from the 39th strain had a WT sequencing length of 702 base pairs. A 127 bp loss in exon3 of another chromosome was visible between the 410th and 537th bases.

**Supplemental Figure 3.** **EFTUD2 regulated functional pathways. (A)** Top20 Biological Process from Gene Ontology (GO) enrichment. **(B)** Top20 functional pathways from Disease Ontology (DO) analysis. **(C)** Top20 functional pathways from Reactome analysis.

**Supplemental Figure 4. Choose the concentration of IFNα-2b.** (A) Effects of IFN on the inhibitive rate of HepG2.2.15 cells. The survival rate of HepG2.2.15 cells was 0.844 ±0.004 at the concentration of 5000 IU/mL IFNα-2b, as analyzed by the CCK8 assay. This indicated that the cytotoxicity on HepG2.2.15 cells is low at 5000 IU/mL IFNα-2b concentration. (B) IFN-α inhibited HBV DNA levels in a dose-dependent manner. HBV DNA in the culture supernatant was measured after nine days of continuous treatment with different concentrations of IFNα-2b (0IU/mL, 500IU/mL, 1000IU/mL, 2000IU/mL, 5000IU/mL). The results revealed that the abundance of HBV-DNA released into the culture medium was significantly decreased dose-dependent. Hence, the concentration of 5000IU/mL was used for the following experiments.

**Supplemental Figure 5.** **Effects of EFTUD2 on cell proliferation.** The CCK8 curve showed that *EFTUD2* single allele knockout suppressed the proliferation capacity of HepG2.2.15 cells. OD450, optical density at 450 nm.

**Supplemental Table 1. List of total gene identified by RNA-Seq differential analysis.** To further investigate the mechanism of EFTUD2 regulating IFN-associated antiviral activity, a transcriptome RNA sequencing was performed in WT and *EFTUD2^+/-^*HepG2.2.15 cells after IFNα-2b treatment. From the mRNA-Seq data, we obtained sequence reads for 20323 genes.

**Supplemental Table 2. List of selected gene for Gene Ontology (GO) analysis, Disease Ontology (DO) analysis and Reactome analysis.** A total of 814 genes showed greater than 2-fold differences (FDR<0.05 and |log2FC|>1), including 439 genes up-regulated and 371 genes down-regulated by *EFTUD2* single allele knockout. These selected gene were used for Gene Ontology (GO) analysis, Disease Ontology (DO) analysis and Reactome analysis.

**Supplemental Table3.** **Supplemental Table3. The difference of spliced transcripts between WT and EFTUD2+/- cells after IFN treatment.** The software rMATS (version 4.0.1) was used to identify differential splicing events between WT and EFTUD2+/-HepG2.2.15 cells after IFNα-2b treatment. The transcripts MX1-201(ENST00000288383), OAS1-202(ENST00000445409) and EIF2AK2-203(ENST00000405334) decreased, and inclusion transcripts like MX1-210(ENST00000467510), OAS1-205(ENST00000549820), EIF2AK2-205 (ENST00000462861) increased in EFTUD2+/-HepG2.2.15 cells.

**Supplemental Figure. 1** **Establish EFTUD2+/-HepG2.2.15 cells.**


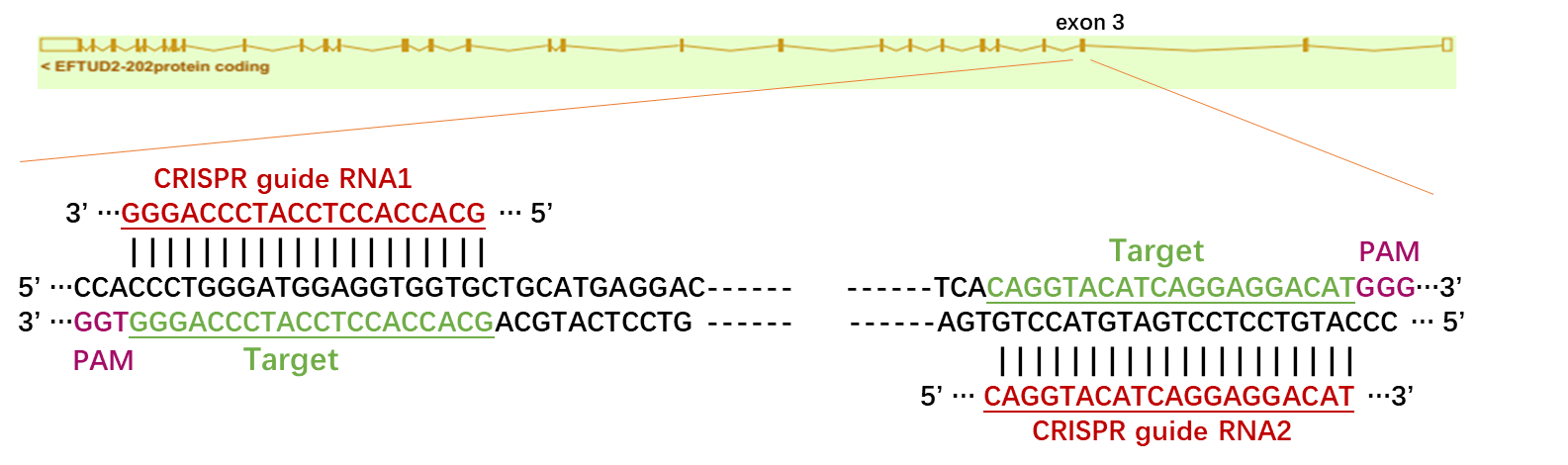
**A.**

**B.**


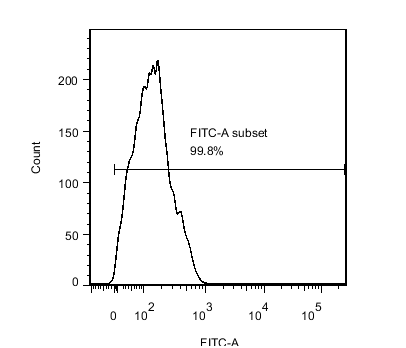


**
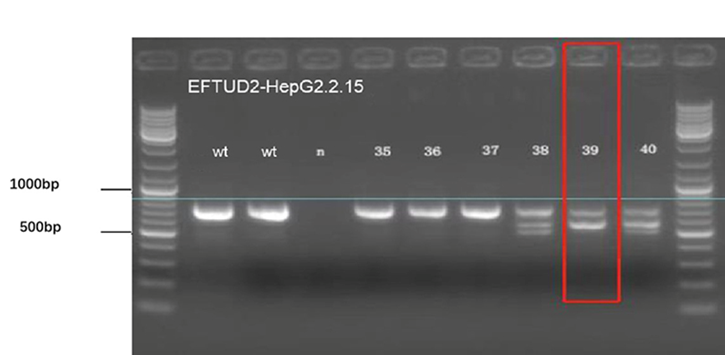
**

**C.**

**Supplemental Figure. 2** **Exon 3 of the EFTUD2 gene has 127bp deletion between sgRNA1 and sgRNA2 cleavage sites.**

**A.**

**
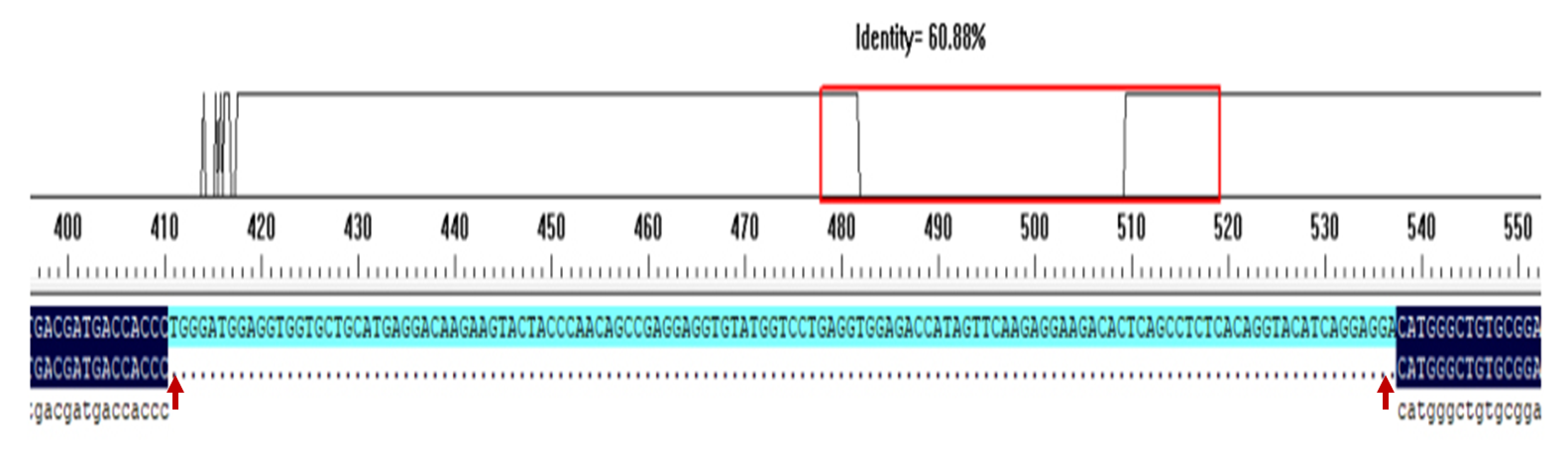
**

**B.**


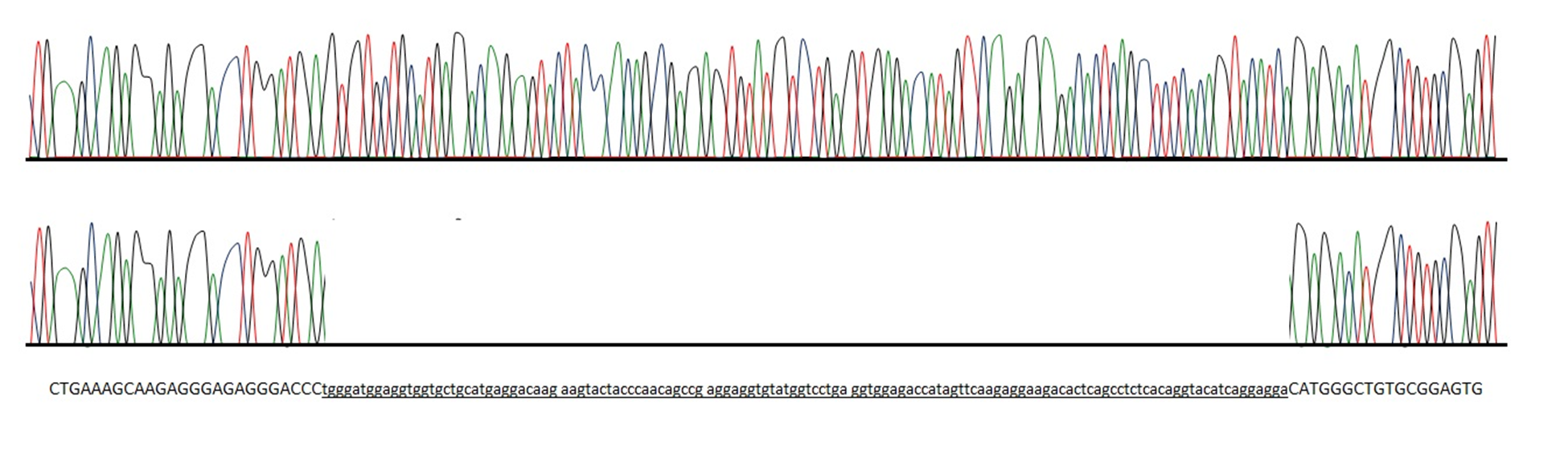


**Supplemental Figure. 3** **EFTUD2 regulated functional pathways.**

**A.**

**
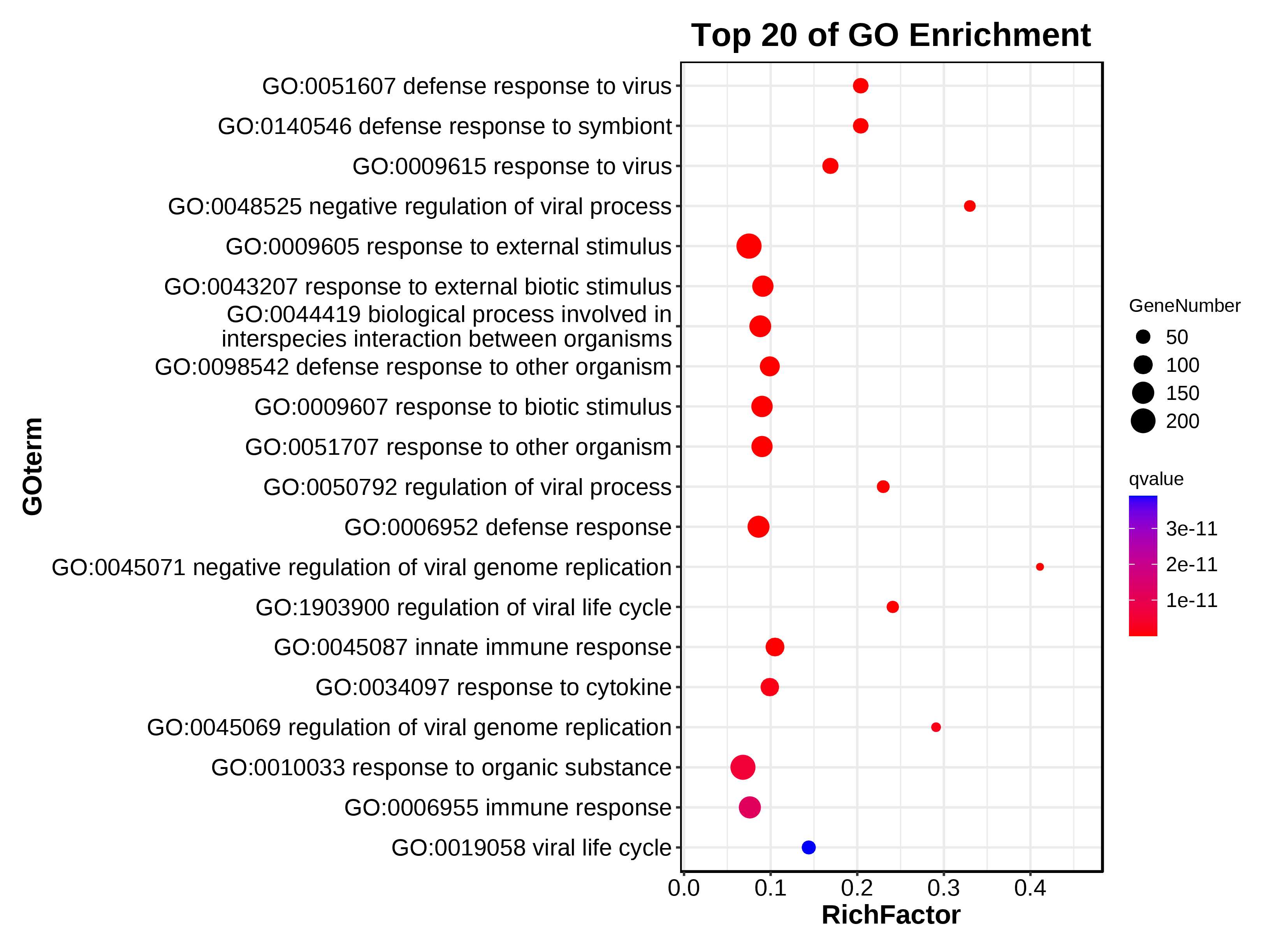
**

**B.**

**
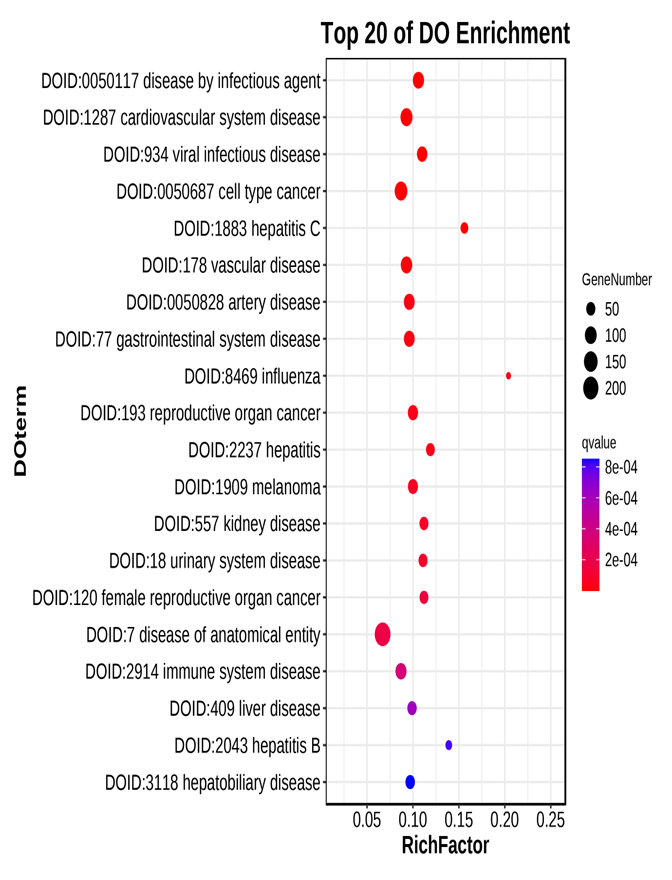
**

**C．**

**
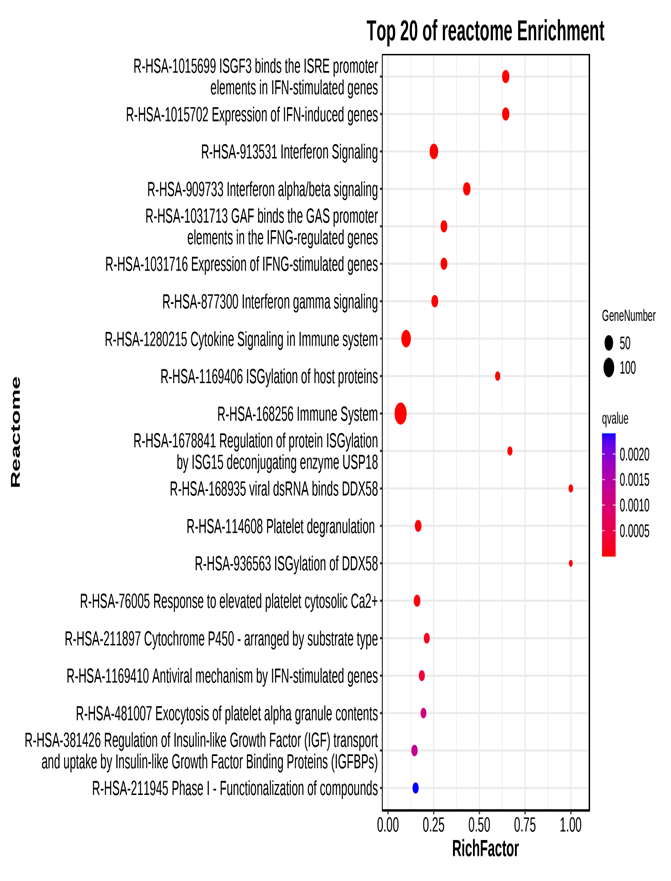
**

**Supplemental Figure. 4** **Choose the concentration of IFNα-2b.**

**A.**

**B.**

**Supplemental Figure. 5** **Effects of EFTUD2 on cell proliferation.**

**A.**
